# CXCR4 chemokine receptor signaling mediates pain in diabetic neuropathy

**DOI:** 10.1186/1744-8069-10-42

**Published:** 2014-06-25

**Authors:** Daniela Maria Menichella, Belmadani Abdelhak, Dongjun Ren, Andrew Shum, Caroline Frietag, Richard J Miller

**Affiliations:** 1 Department of Neurology, Robert Lurie Medical Research Center, Northwestern University, Lurie 8-123, 303 E. Superior St, Chicago, IL, USA; 2 Department of Molecular Pharmacology, Northwestern University, Chicago, IL, USA

**Keywords:** Chemokine, Neuropathic pain, Painful diabetic neuropathy, DRG neurons

## Abstract

**Background:**

Painful Diabetic Neuropathy (PDN) is a debilitating syndrome present in a quarter of diabetic patients that has a substantial impact on their quality of life. Despite this significant prevalence and impact, current therapies for PDN are only partially effective. Moreover, the cellular mechanisms underlying PDN are not well understood. Neuropathic pain is caused by a variety of phenomena including sustained excitability in sensory neurons that reduces the pain threshold so that pain is produced in the absence of appropriate stimuli. Chemokine signaling has been implicated in the pathogenesis of neuropathic pain in a variety of animal models. We therefore tested the hypothesis that chemokine signaling mediates DRG neuronal hyperexcitability in association with PDN.

**Results:**

We demonstrated that intraperitoneal administration of the specific CXCR4 antagonist AMD3100 reversed PDN in two animal models of type II diabetes. Furthermore DRG sensory neurons acutely isolated from diabetic mice displayed enhanced SDF-1 induced calcium responses. Moreover, we demonstrated that CXCR4 receptors are expressed by a subset of DRG sensory neurons. Finally, we observed numerous CXCR4 expressing inflammatory cells infiltrating into the DRG of diabetic mice.

**Conclusions:**

These data suggest that CXCR4/SDF-1 signaling mediates enhanced calcium influx and excitability in DRG neurons responsible for PDN. Simultaneously, CXCR4/SDF-1 signaling may coordinate inflammation in diabetic DRG that could contribute to the development of pain in diabetes. Therefore, targeting CXCR4 chemokine receptors may represent a novel intervention for treating PDN.

## Background

Neuropathic pain in diabetes, Painful Diabetic Neuropathy (PDN), is a debilitating affliction present in 26% of diabetic patients [1-3] with substantial impact on their quality of life [4]. Despite this significant prevalence and impact, current therapies for PDN are only partially effective. Moreover, the molecular and electrophysiological mechanisms underlying PDN are not well understood.

This lack of understanding of the pathogenesis and the molecular mechanisms underlying neuropathic pain represents a barrier to further progress in this field as emphasized by the failure of numerous potential therapeutic approaches to successfully treat PDN. Opiates are not particularly effective in treating neuropathic pain and given the chronic nature of this syndrome their use is problematic [3]. Other drugs, such as gabapentinoids and antidepressants, do produce limited relief in some patients but the presence of significant side effects and their lack of effectiveness in many patients [5,6] means that better therapeutic approaches are urgently needed. Given the prevalence of PDN and the absence of effective therapies we wanted to elucidate the molecular and physiological mechanisms responsible for PDN as a critical step towards developing more effective targeted therapeutic interventions in this disorder.

Pain is a physiological response to potentially dangerous noxious stimuli. However, pathological or “neuropathic pain” is associated with sustained excitability of sensory neurons within pain pathways leading to reduced nociceptive thresholds and the development of pathological spontaneous activity, so that pain is produced in the absence of appropriate stimuli [7-9]. The molecular mechanisms responsible for abnormal excitability in sensory neurons leading to neuropathic pain are mostly unknown. However, Dorsal Root Ganglia (DRG) sensory neurons develop pathological spontaneous activity in response to different molecules such as inflammatory cytokines and chemokines [10-14].

Chemokines, or chemotactic cytokines, are a large group of proteins important for the regulation of leukocyte migration [15,16]. In the last decade, numerous studies have demonstrated that, in addition to their role in coordinating the immune response, chemokines have important functions in the nervous system [17]. For example, excitatory chemokine signaling has been implicated in the pathogenesis of neuropathic pain in several animal models [18]. In particular, some reports have implicated the chemokine stromal cell derived factor 1 (SDF-1) and its receptor CXCR4 in the pathogenesis of neuropathic pain in a subset of animal models including HIV-1 induced neuropathy [19,20] and opiate induced hyperalgesia [21]. Importantly, CXCR4 receptor expression as measured by microarray analysis was increased in peripheral nerve samples from diabetic patients with progressive diabetic neuropathy [22], suggesting a similar role for CXCR4 receptors in PDN.

Based on this evidence, we hypothesized that excitatory CXCR4/SDF-1 signaling in DRG neurons may play a critical role in the pathogenesis of PDN.

We now demonstrate that administration of a selective CXCR4 antagonist, AMD3100, substantially reverses neuropathic pain in animal models of type II diabetes. These results indicate that CXCR4 mediated signaling is necessary for PDN in mice. This hypothesis is consistent with the expression of CXCR4 by a subpopulation of DRG neurons and increased excitatory effects of the chemokine SDF-1 on diabetic DRG neurons in culture. Additionally, we observed numerous CXCR4 expressing inflammatory cells infiltrating the DRG of diabetic mice.

Overall, these data suggest that CXCR4/SDF-1 signaling mediates enhanced calcium influx and excitability in DRG neurons responsible for PDN. CXCR4/SDF-1 signaling may simultaneously coordinate inflammation in diabetic DRG that could contribute to the development of pain in diabetes. Therefore, CXCR4 signaling may constitute a novel target for therapeutic intervention to PDN.

## Results

### The specific CXCR4 antagonist AMD3100 reverses neuropathic pain in two animal models of type-II diabetes

**Figure 1:**
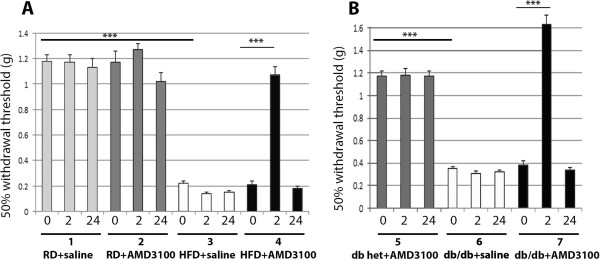
**The specific CXCR4 antagonist, AMD3100, reversed neuropathic pain in type II diabetic mice. A**. Experimental animals: (1) wild type (wt) mice on Regular Diet (RD) injected with saline (n = 8), (2) wt on RD injected with CXCR4 antagonist, AMD3100 (n = 8), (3) Diabetic wt mice on High Fat Diet (HFD) injected with saline (n = 8); (4) Diabetic wt on HFD injected with AMD3100 (n = 8). Pain behavior was assessed using Von Frey test prior (0), 2hrs (2) and 24 hrs (24) after antagonist or saline injection. In diabetic wt mice on HFD (3) prior to antagonist injection the withdrawal threshold was significantly reduced compared with wt on RD (1) demonstrating neuropathic pain in HFD induced diabetes. Following CXCR4 antagonist injection in wt HFD (4) at 2hrs after injection (2) paw withdrawal threshold increases significantly demonstrating that blocking CXCR4 receptors reversed neuropathic pain in HFD induced diabetic mice. At 24hrs (24) this effect was absent. Wt mice on HFD injected with saline (3) did not show any change. **B**. Experimental animal: (5) Control db-db heterozygous (db het) mice injected with AMD3100 (n = 6), (6) Diabetic db-db homozygous (db/db) mice injected with saline (n = 8) (7) Diabetic db/db mice injected with AMD3100 (n = 8). In diabetic db/db mice (6) prior to antagonist injection the withdrawal threshold was significantly reduced compared with control db het mice, demonstrating neuropathic pain in the db/db model of type II diabetes. Following CXCR4 antagonist injection in diabetic db/db mice (7) at 2hrs after injection (2) paw withdrawal threshold increases significantly, demonstrating that blocking CXCR4 receptor reverse neuropathic pain in db-db model of type II diabetes. Db het control mice injected with AMD3100 (5) did not show any change. Values are expressed as mean+/− S.E. (p < 0.001).

In our first series of experiments we set up a model of type II diabetes by feeding mice with a High Fat Diet (HFD) for 10 weeks. These mice exhibited a gain in weight and became glucose intolerant indicating the development of diabetes (Table 1). Mice fed with a regular diet (RD) did not develop diabetes. In order to examine whether HFD diabetic mice also displayed signs of pain hypersensitivity, pain behavior was assessed using Von-Frey filaments. We observed that in HFD diabetic mice the withdrawal threshold required to elicit a response was significantly reduced compared with RD non-diabetic mice, demonstrating the development of neuropathic pain. Statistical analysis revealed that the 50% threshold was 0.223 gram (g) with SE 0.015 in HFD mice (n = 8) compared to 1.182 g with SE 0.048 in RD mice (n = 8) (p < 0.001) (Figure 1).

**Table 1:** Glucose tolerance test (GTT) and weight of wild-type mice on high-fat-diet (WT HFD) and wild-type mice on regular diet (WT RD)

**Experimental animals**	**Weight (g)**	**Fasting glucose (mg/dl)**	**30 mins GTT (mg/dl)**	**60 mins GTT (mg/dl)**	**120 mins GTT (mg/dl)**
**WT HFD**	**34.0 +/− 0.8**	**112.2 +/− 4.7**	**465.1 +/− 13.9**	**444.9 +/− 18.0**	**264.7 +/− 14.0**
**WT RD**	**26.9 +/− 0.4**	**89.3 +/− 4.4**	**217.0 +/− 17.9**	**188.5 +/− 4.4**	**161.3 +/− 8.5**

Value is expressed as mean+/− S.E.; p-value determined by two tailed Student’s t-testing with significance at p<.001.

In order to determine whether SDF-1/CXCR4 signaling played a role in this behavior we examined the effects of blocking CXCR4 receptors on pain behavior in this model of type II diabetes. The highly selective CXCR4 chemokine receptor antagonist AMD3100 was administered at a concentration of 5 mg/kg, given as a single intraperitoneal (i.p.) injection. We elected to use this concentration of AMD3100 as we have previously demonstrated it to be effective in other animal models of neuropathic pain [19,20]. Von Frey behavioral studies for mechanical allodynia were repeated at 2 and 24 hours after AMD 3100 administration. These times points were elected as we had already demonstrated that AMD3100 effectively reversed neuropathic pain in other animal models one hour after intraperitoneal injection [19,20].

These experiments demonstrated that AMD3100 substantially reversed neuropathic pain in HFD diabetic mice. Indeed, 2 hours after antagonist injection in HFD diabetic mice the paw withdrawal threshold increased significantly compared with HFD induced diabetic mouse injected with saline. Statistical analysis revealed that the 50% threshold was 1.001 g with SE 0.07 in HFD diabetic mice injected with ADM3100 (n = 8) compared to 0.157 g with SE 0.013 in HFD diabetic mice injected with saline (n = 8). Values are expressed as means +/− SE (p <0.001) (Figure 1A). After 24 hours this effect was no longer apparent (Figure 1A). In contrast a selective CCR2 chemokine receptor blocking drug only minimally reversed allodynia in HFD induced diabetic mice. Statistical analysis revealed that the 50% threshold was 0.302 g with SE 0.024 in HFD diabetic mice injected with CCR2 antagonist (n = 6) compared to 0.174 g with SE 0.019 in HFD diabetic mice injected with saline (n = 8). Values are expressed as means +/− SE (p <0.001). This result differs from some other pain models our laboratory has investigated such as in osteoarthritis where CCR2 receptor deletion or CCR2 receptor antagonists are much more effective in ameliorating pain [23].

In order to confirm our findings in an additional model of type II diabetes we repeated the same set of experiments in a second well characterized model in which mice carry a leptin receptor null mutation: the C57BLKS db/db (db/db) mouse line. Db/db mice are known to display mechanical allodynia after 6 weeks of age [24]. In keeping with these previous reports, we observed that 18 weeks old homozygous db/db mice displayed tactile allodynia (Figure 1B). Von Frey behavioral studies for mechanical allodynia were repeated at 2 and 24 hours after AMD3100 administration at a concentration of 5 mg/kg, given as a single intraperitoneal injection (i.p.). These experiments demonstrated that AMD3100 also substantially reversed neuropathic pain in homozygous db/db mice at 2 but not 24 hours after administration of the drug. Statistical analysis revealed that the 50% threshold was 1.637 g with SE 0.084 in db/db injected with AMD3100 (n = 8) compared with 0.320 g with SE 0.021 in db/db mice injected with saline (n = 8). Values are expressed as means +/− SE (p <0.001) (Figure 1B).

Overall this first series of experiments demonstrated that SDF-1/CXCR4 signaling is necessary for the expression of pain hypersensitivity in two separate animal models of type II diabetes. As a result of these observations we next wanted to investigate the status of excitatory CXCR4/SDF-1 signaling in DRG neurons taken from diabetic animals.

### Enhanced SDF-1 induced increases in intracellular calcium concentrations [Ca2+]i in HFD induced diabetic DRG neurons

**Figure 2:**
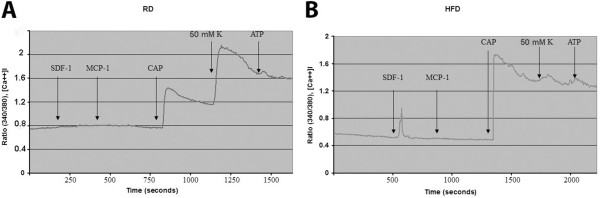
**Application of the chemokine SDF-1 increased intracellular calcium concentration in DRG sensory neurons in HFD-induced diabetes.** Representative trace of intracellular calcium responses of acutely cultured DRG sensory neurons from RD mice **(A)** and HFD mice **(B)** indicating a response to SDF-1 in diabetic DRG. Response to high K + (50 mM) and to capsaicin as positive control is also shown in RD (A) and HFD DRG (B).

One way that chemokine signaling could contribute to chronic pain is by directly exciting DRG neurons, something that is associated with increases in neuronal [Ca2+]i [25]. Our laboratory and others have already shown that activation of CXCR4 receptors by its cognate ligand, the chemokine SDF-1, results in excitation of DRG sensory neurons and in increased [Ca2+]i in some animal models of neuropathic pain [11]. Using [Ca2+]i imaging it is possible to test for the presence of functional receptors as well as other properties of the neurons concerned. We observed that application of SDF-1 increased [Ca2+]i in DRG sensory neurons from HFD mice (Figure 2B), although it did so much less frequently in non-diabetic animals on a regular diet (Figure 2A). Statistical analysis revealed that upon application of SDF-1, 89 ± 3.766% of DRG neurons from HFD diabetic mice increased [Ca2+]i (n = 140) compared to 27 ± 2.680% in RD (n = 140). Values are expressed as means ± SE% (p <0.001).In all experiments Monocyte Chemoattractant Protein-1 (MCP-1) was also applied. However, the percentage of DRG sensory neurons responding to MCP-1 was not statistically different in RD and HFD mice (data not shown). For all experiments capsaicin, high K + (50 mM) and ATP were added to assess the cell’s identity and viability, respectively. A response to high K + stimulation indicates the presence of voltage dependent Ca2+ channels, which is indicative of neurons. Additionally, a positive response to capsaicin as well as high K + indicates the cell is a nociceptor of the subtype expressing the TRPV1 channel. A response to ATP, which activates purinergic receptors, in the absence of a response to high K + and/or capsaicin, indicates a non-neuronal cell, presumably a type of glial cell such as a satellite glial cell or Schwann cell. According to these criteria all of the cells that responded to either SDF-1 or MCP-1 were neurons. (Figure 2).

These results indicate that a increased number DRG neurons express functional CXCR4 receptors in diabetic mice and suggest that increased excitatory CXCR4/SDF-1 signaling in DRG neurons could contribute to PDN.

### CXCR4/SDF-1 signaling in diabetic DRG sensory neurons

**Figure 3:**
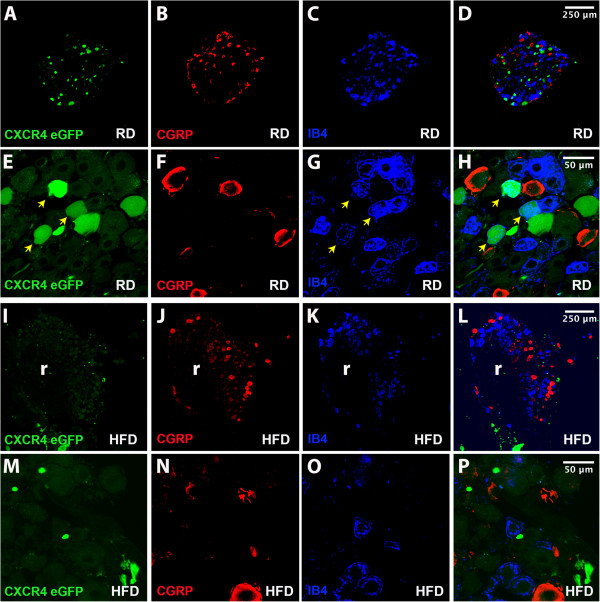
**CXCR4 expression patterns in DRG sensory neurons. A**-**H**: Confocal analysis of DRG isolated from non-diabetic CXCR4-eGFP mice fed with regular diet (RD) showing co-localization of CXCR4-eGFP in green with either CGRP (red) (B and F) or IB4 (purple) (C and G). The majority of DRG sensory neurons expressing CXCR4-eGFP are IB4 positive (C and G, arrows). **Quantification:** the percentage of CXCR4-eGFP positive cells expressing IB4 was 32.8 ± 6.5% (n = 9) compared to the percentage of CXCR4-eGFP positive cells expressing CGRP 1.78 ± 1.78% (n = 7). Values are expressed as means +/− SE% (p <0.001). **I-P:** Confocal analysis of DRG isolated from diabetic CXCR4-eGFP mice fed with High Fat Diet (HFD). CXCR4-eGFP reporter gene transcriptional activity appeared to be decreased in HFD induced diabetic DRG (I-P). **Quantification:** the percentage of IB4 positive DRG expressing CXCR4-EGFP in RD was 32.8 ± 6.5% (n = 9) compared to 1.0 ± 0.73% (n = 9) in HFD mice. Similarly, the percentage of CGRP positive DRG neurons expressing CXCR4-eGFP was also reduced in HFD-induced mice compared to RD non diabetic mice. Indeed, the percentage of CGRP positive DRG expressing CXCR4-EGFP in RD was 1.78 ± 1.78% (n = 7) compared with the 0.42 ± 0.42 (n = 9) in HFD DRG neurons. Values are expressed as means ± SE% (p <0.001). (Magnification 20x (scale bar 250 μm) in A-D and I-L; magnification 40x (scale bar 50 μm) in E-H and M-P). r: dorsal root.

Extensive characterization of DRG neurons has revealed the existence of molecularly and functionally defined subpopulations of cells. According to one type of classification following sensory neurogenesis prospective DRG nociceptive neurons undergo two distinct differentiation pathways leading to the formation two major classes of molecularly defined DRG neurons: 1) TrKA positive sensory neurons, also known as “peptidergic” neurons which express Calcitonin Gene Related Peptide (CGRP) or Substance P and 2) Ret positive sensory neurons also known as “non-peptidergic” neurons, first identified for their ability to bind isolectin IB4 [26]. These two sets of DRG sensory neurons express distinct populations of ion channels and receptors [26,27] and carry different pain modalities [27,28]. Therefore, it is important to determine which subpopulation of molecularly defined DRG neurons expresses CXCR4 receptors.In order to define CXCR4 expression by DRG neurons, we used mice in which the fluorescent protein eGFP is expressed under the control of the CXCR4 promoter (CXCR4-eGFP mice). Confocal analysis of DRG neurons in non-diabetic CXCR4-eGFP mice showed that CXCR4-eGFP was expressed in a subset of non-peptidergic (IB4 positive) but not in peptidergic (CGRP positive) DRG neurons (Figure 3A-H). Interestingly, CXCR4-eGFP reporter gene transcriptional activity was clearly decreased in HFD induced diabetic DRG (Figure 3I-P). Indeed, the percentage of IB4 positive DRG neurons expressing CXCR4-eGFP was dramatically reduced in HFD induced mice compared to RD non-diabetic mice (quantification in figure legend, Figure 3I-P).

**Figure 4:**
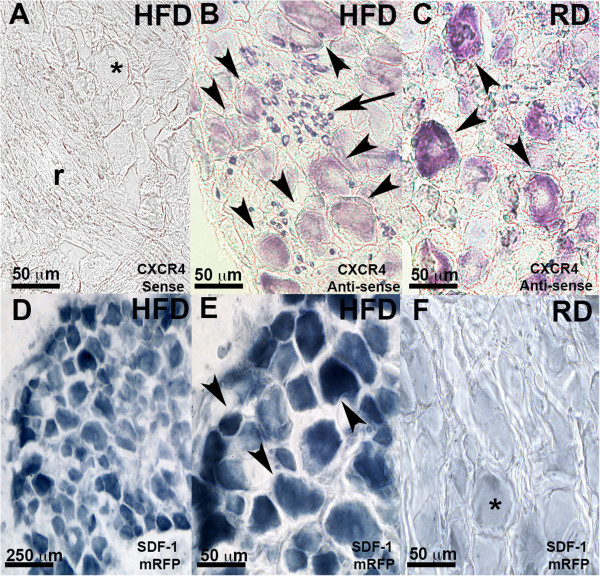
**CXCR4 and SDF-1 expression in diabetic mouse DRG. A**-**C**: Representative images of *in situ* hybridization experiments using an antisense probe for CXCR4 receptors on DRG sections taken from diabetic mice fed with HFD **(B)** or control non-diabetic mice fed with RD **(C)**. Arrows indicate CXCR4 positive immune cells infiltrating the diabetic DRG. Arrowheads indicate neurons expressing CXCR4 chemokine receptors. In HFD DRG expression of CXCR4 is in more neurons but at reduced levels per cells **(B)** compared to control DRG **(C)**. **Quantification:** 15.652 ± 1.55 cells expressing CXCR4 mRNA in HFD DRG compared with 7.833 ± 1.489 cells expressing CXCR4 mRNA in RD DRG. Values are expressed as means ± SD (p <0.001). Sense probe control is shown for HFD conditions **(A)**, * indicates DRG neurons, r indicates dorsal root. **D**-**F**: DRG from SDF-1- mRFP mice diabetic fed with HFD **(D and E)** or non-diabetic mice fed with RD **(F)**. Immunolabeling for mRFP reveals SDF-1 expression in neurons (arrowheads) in HFD induced diabetic mice **(D and E)**. E is higher magnification of panel in D. Expression of SDF-1-mRFP in diabetic mice was observed in numerous neurons of many different sizes whereas only a few neurons exhibited low levels of chemokine expression in normal mice. **Quantification:** 42.828 ± 8.05 cells expressing SDF-1 mRFP in HFD DRG (n = 3 animals, 10 sections from each animal) compared with 1.710 ± 1.21 cells expressing SDF-1 mRFP in RD DRG (n = 3 animals, 10 sections from each animal). Values are expressed as means ± SD (p <0.001). Asterisk (*) indicates lack of neuronal SDF-1-mRFP expression in RD fed mice **(F)**. (Magnification 20x (scale bar 250 μm) in **D**; magnification 40x (scale bar 50 μm) in **A**, **B**, **C**, **E**, **F**).

The expression of CXCR4 receptors was also investigated using *in situ* localization of CXCR4 mRNA. These experiments showed that some DRG neurons normally exhibited very high levels of CXCR4 mRNA (Figure 4C). *In situ* micrographs demonstrated that very few neurons from HFD animals expressed high levels of CXCR4 mRNA. Interestingly, however, there was an increased number of neurons in HFD animals that expressed lower levels of CXCR4 receptor mRNA (Figure 4B). Quantification of DRG neurons expressing CXCR4 receptors revealed that overall the total number of DRG neurons expressing CXCR4 mRNA actually increased in diabetic conditions compared to non-diabetic DRG neurons (quantification in figure legend, Figure 4A-C).

We also examined the state of SDF-1 expression in the DRG from normal and diabetic animals. We elected to assess SDF-1 expression using mice expressing an SDF-1-mRFP transgene generated in our laboratory [29]. Using an antibody against mRFP, we observed that DRG neurons from HFD diabetic SDF-1-mRFP mice greatly increased their expression of SDF-1 (Figure 4D and E) compared to DRG neurons from RD mice (Figure 4F) (quantification in figure legend, Figure 4D-F).

Hence, SDF-1 released from DRG neurons would be an ideal position to activate excitatory CXCR4 receptors expressed by DRG neurons in PDN.

### Inflammatory cell infiltration into diabetic DRG

**Figure 5:**
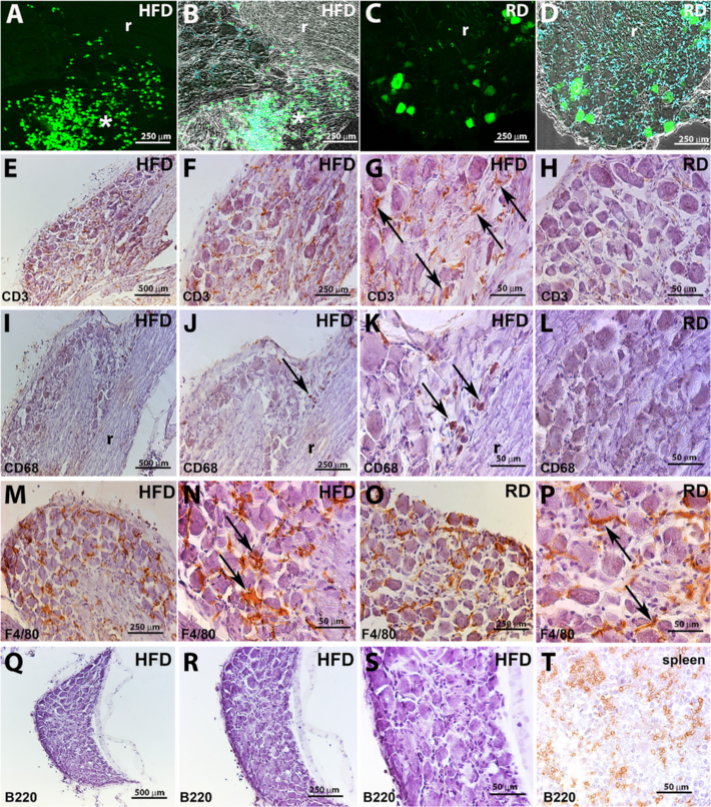
**CXCR4 positive inflammatory cells infiltrate diabetic DRG.** Large influx of CXCR4-eGFP positive cells into HFD diabetic mice DRG (**A** and **B**, asterisk). In contrast virtually no cells were observed in non-diabetic RD DRG (**C** and **D**). **Quantification:** 79.131 ± 15.03 CXCR4 expressing inflammatory cells infiltrating into HFD DRG compared to 1.363 ± 0.82 in RD DRG. Characterization of the nature of the inflammatory infiltrate revealed the presence of CD3 positive T-cells in diabetic DRG (**E**-**G**, arrows). On the contrary virtually no CD3 positive T-cells were noted in non-diabetic DRG **(F)**. Quantification: 39.132 ± 11.14 CD3 positive cells in HFD DRG compared to 1.945 ± 0.84 in RD DRG. Interestingly, cells infiltrating the diabetic DRG at the dorsal root entry (arrows) were CD68 positive macrophages **(I-K)**. Virtually no CD68 positive cells were noted in non-diabetic DRG **(L)**. Quantification: 16.333 ± 3.15 CD68 positive cells in HFD DRG compared to 0.205 ± 0.41 in RD DRG. The number of F4/80 positive macrophages in diabetic DRG (**M** and **N**) was the same compared to non-diabetic DRG (**O** and **P**). **Quantification:** 26.615 ± 2.48 F4/80 positive cells in HFD DRG compared to 26.558 ± 2.72 cells in RD DRG. However, F4/80 positive macrophages exhibited an altered morphology in diabetic DRG (**N**, arrows) compared with non-diabetic DRG (**P**, arrows). Indeed, F4/80 positive cells in diabetic DRG assumed the morphology of activated macrophages (**N**, arrows). No B220 positive B-cells were noted in diabetic DRG **(Q-S)** using an antibody against B220 able to visualize B-cells in the spleen **(T)**. (Magnification 10X, scale bar 500 μm: **E**, **I**, **Q**; 20x, scale bar 250 μm: **A**-**D**, **F**, **J**, **M**, **O**, **R**; 40X scale bar 50 μm: **G**, **H**, **K**, **L**, **N**, **P**, **S**, **T**). r: dorsal root. Values expressed as means ± SD (p <0.001).

In addition to the cell bodies of sensory neurons, DRGs contain satellite glial cells, resident macrophages and it has also been shown that they may contain infiltrating leukocytes under inflammatory conditions [30,31]. All of these cell types might potentially contribute to pain signaling [25,28,31]. Chemokine signaling can promote inflammatory infiltration into the spinal cord and DRG following nerve injury and in other models of neuropathic pain [10,30]. We observed numerous CXCR4 expressing inflammatory cells infiltrating into HFD diabetic DRG (Figure 5A and B). Characterization of the nature of the inflammatory infiltrate revealed the presence of CD3 positive T-cells in HFD DRG (Figure 5E-G). In contrast, virtually no CD3 positive T-cells were noted in control non-diabetic DRG (Figure 5H). Interestingly, cells infiltrating into the diabetic DRG perineurium at the site of the dorsal root entry zone were mainly CD68 positive macrophages (Figure 5I-K). Virtually no CD68 positive cells were noted in control no diabetic RD DRG (Figure 5L). In contrast, the number of F4/80 positive macrophages in DRG under diabetic conditions (Figure 5M and N) was the same compared to control non-diabetic RD DRG (Figure 5O and P). However, F4/80 positive macrophages exhibited an altered morphology in diabetic DRG (Figure 5N) compared with non-diabetic RD DRG (Figure 5P). F4/80 positive cells in diabetic DRG assumed the morphology of activated macrophages (Figure 5N). Finally, we did not observe B220 positive B-cells in HFD-induced diabetic DRG or in RD non-diabetic DRG (Figure. 5Q-S) using an antibody against B220 which was clearly able to visualize B-cells in the spleen (T). (Quantification in figure legend, Figure 5).

The presence of CXCR4 expressing immune cells with the prevalence of CD3 positive T-cells in diabetic DRG may have important implications for the generation of pain in diabetes.

## Methods

### Animals

All animal experiments were approved by the Institutional Animal Care and Use Committee at Northwestern University. Animals were housed with food and water ad libitum and kept on 12-hours light cycle. Wild-type 8 weeks-old male C57BL/6 (Jackson Laboratories), 18 weeks old db-db homozygous mice (Homozygous Lepr db, BKS.Cg-*Dock7*^*m*^ −/− *Lepr*^*db*^/J, Jackson Laboratories), 18 weeks old heterozygous mice (Heterozygous Lepr db, BKS.Cg-*Dock7*^*m*^ +/− *Lepr*^*db*^/J, Jackson Laboratories), 8 weeks old CXCR-4 eGFP mice and SDF-1-mRFP mice, generated in our laboratory [29], were used in these studies.

### High Fat Diet (HFD)-induced diabetes as a model of Diabetes type II

HFD induces insulin resistance and is therefore used to create rodent models of diabetes type-II [32]. Wild-type 8 weeks-old male C57BL/6 mice, CXCR-4 eGFP mice and SDF-1-mRFP mice were fed with a 42% fat diet for 10 weeks. Control mice were fed with regular diet (11% fat diet). After 10 weeks on HFD, Glucose Tolerance Test (GTT) was performed as previously described [32]. Briefly, mice, after fasting for 12 hours, were injected with a 45% D-glucose solution (2mg glucose/1g animal body weight). Blood glucose is measured immediately before and 30, 60, and 120 minutes after glucose injection using an OneTouch UltraMini glucose meter and OneTouch Ultra test strips.

### Behavioral testing

Von Frey behavioral studies were performed as previously described [9,19,20,33,34]. Briefly, mice were placed on a metal mesh floor and covered with a transparent plastic dome where the animal rested quietly after an initial few minutes of exploration. Animals were habituated to this testing apparatus for 15 minutes a day, two days prior to pre-injection behavioral testing. Following acclimation, each filament was applied to the glabrous side of the hind paw. Mechanical stimuli were applied with seven filaments, each differing in the bending force delivered (10, 20, 40, 60, 80, 100, and 120 mN), but each fitted a flat tip and a fixed diameter of 0.2 mm. The force equivalence of mN to grams is: 100 mN = 10.197 grams. The filaments were tested in order of ascending force, with each filament delivered for 1 second. The interstimulus interval was 10–15 seconds [34].

### Statistical analysis

The incidence of foot withdrawal was expressed as a percentage of six applications of each filament as a function of force. A Hill equation was fitted to the function (Origin version 6.0, Microcal Software) relating the percentage of indentations eliciting a withdrawal to the force of indentation. From this equation, the threshold force was obtained and defined as the force corresponding to a 50% withdrawal rate [34]. The GB-Stat School Pack software (Dynamic Microsystem) was used to statistically evaluate all data. The one-way analysis of variance (ANOVA) with a Dunnett’s Multiple Comparison test was used to analyze difference between multiple experimental groups. A probability level of .05 indicates significance.

### CXCR4 chemokine receptor blocker administration

The highly specific CXCR4 antagonist, AMD3100 (Sigma (St Louis, MO, USA), was used for the all the experiments. The CXCR4 chemokine receptor antagonist was administered to HFD induced diabetic and control non-diabetic mice 10 weeks after diet, to 18 weeks old diabetic homozygous C57BLKS db/db (db/db) mice and control heterozygous C57BLKS db/db (db het) mice. The drug was freshly prepared in saline on the day of the experiment at a concentration of 5 mg/kg and given as a one-time intraperitoneal (i.p.) injection.

### Preparation of acutely cultured dorsal root ganglion neurons

DRG sensory neurons from HFD-induced diabetic wild-type mice were acutely dissociated as previously described [20] after 10 weeks of HFD. Wild type mice on regular diet were used as controls. Briefly, DRG neurons were acutely dissociated via collagenase 4 (1 μg/ml) and papain (30 U/ml), Worthington Biochemical Corp, Lakewood, NJ) digestion. Cells were plated on poly-L-lysine and laminin (20 μg/ml) coated glass coverslips and cultured at 37 degrees Celsius with 5% CO2, for 48 hrs in adult neurogenic medium: F12 with L-glutamine, 0.5% FBS, 1xN2 (Life Techonologies), penicillin (100 μg/ml) and streptomycin (100 U/ml).

### Intracellular calcium imaging in vitro

The response of acutely cultured DRG neurons to the chemokines SDF-1 and MCP-1 (500 ng/ml R&D Systems) were recorded using intracellular calcium imagining following a standard protocol previously used in our laboratory [20]. Briefly, acutely cultured DRG cells were loaded with fura-2 AM (3uM, Invitrogen, Carlsbad, CA) for 25 minutes at room temperature in a balanced salt solution (BSS) [NaCl (140 mM), Hepes (10 mM), CaCl2 (2 mM), MgCl2 (1 mM), Glucose (10 mM), KCl (5 mM)]. The cells were rinsed with the BSS and mounted onto a chamber that was placed onto the inverted microscope and continuously perfused with BSS at a rate of 2 ml/min. Intracellular calcium ([Ca2+]i) was measured by digital video microfluorometry with an intensified CCD camera coupled to a microscope and MetaFluor software. Cells were illuminated with a 150W xenon arc lamp, and the excitation wavelengths of the fura-2 (340/380 nm) were selected by a filter changer. Chemokines were applied for two minutes directly into the coverslip bathing solution after the perfusion was stopped. If no response was seen within 1 minute, the chemokine was washed out. For all experiments, SDF-1 (500 ng/ml, R&D Systems) and MCP-1 (500 ng/ml, R&D Systems), capsaicin (100 nM), high K + (50 mM) and ATP (100 μM) were added to the cells.

### Statistical analysis

The significance of differences between the control group, and the various treatment groups were statistically analyzed. Within each treatment group, post-drug administration means were compared with the baseline/control values by analysis of variance (ANOVA).

### Immunohistochemical labeling: Immunofluorescence

Adult CXCR4-eGFP mice were deeply anesthetized with isoflurane and transcardially perfused with saline followed by 4% paraformaldehyde. Lumbar ganglia were removed and post fixed for 4 hours. Sagittal sections of the DRG were serially cut at 20 μm onto Super Frost microscope slides (Fisher Scientific, Pittsburgh PA). At least 6 sections were obtained for immunocytological analysis per DRG. Slides were incubated with blocking buffer (3% BSA/3% horse serum/0.4% Triton-X; Fisher Scientific, Pittsburgh PA) for 1 hour, followed by overnight incubation with primary antibody. We used the following antibodies on CXCR4-eGFP DRG section: I-isolectin B4 (IB4-conjugated 647 from Invitrogen, used at 1:100 dilution) and Calcitonin gene-related peptide (CGRP rabbit polyclonal Ab from Abcam, used at 1:4000). Slides were washed in PBS three times for 5 minutes each and cover slipped with a Vectasheld.

### Confocal analysis

Tissue sections were analyzed by confocal microscopy for co-expression of CXCR4 chemokine receptor eGFP and Isolectin (IB4) or Calcitonin gene-related peptide (CGRP) using confocal microscopy.

### Statistical analysis

The proportions of immune-reactive neurons were determined from the total number of CXCR4-eGFP positive neuron present in a tissue section. At least 5000 neuronal profiles from six animals (minimum of 625 cells per ganglia) were quantified for each cell type in the single neuronal marker study and for each combination of cellular markers. Data are represented as means ± SE%.

### Peroxidase-diaminobenzidine (DAB) immunolabeling

Wild-type 8 weeks-old male C57BL/6 mice and 8 weeks-old male SDF-1-mRFP mice, HFD-induced diabetic and control RD non-diabetic mice were deeply anesthetized with isoflurane and trans-cardially perfused with saline followed by 4% paraformaldehyde. Lumbar ganglia were removed and post fixed for 4 hours. Sagittal sections of the DRG were serially cut at 20 μm onto Super Frost microscope slides (Fisher Scientific, Pittsburgh PA). At least 6 sections were obtained for immune-cytological analysis per DRG. Sections were incubated with 3% hydrogen peroxidase for 30 minutes at room temperature to block endogenous peroxidase. Slides were then incubated with blocking buffer (5% donkey serum) for 1 hour, followed by overnight incubation with primary antibody. We used the following antibodies on DRG sections: anti-CD3 rabbit polyclonal antibody from Dako, used at 1:500; anti-CD68 rabbit polyclonal antibody from Abcam, used at 1:500; anti-F4/80 rat antibody from eBiosciences, used at 1:500 and anti-B220 rat antibody from BD Biosciences, used at 1:1000. Slides were washed three times in PBS for 5 minutes each and then incubated with secondary biotinylated antibody for 1 hour at room temperature. Colorimetric reaction was carried out using the ABC kit and streptavidin-HRP and DAB system from Vector Laboratories. Additionally, we used antibodies against RFP on DRG from SDF-1-RFP mice to detect chemokine SDF-1 (anti-RFP rabbit polyclonal Ab from Abcam, used at 1:1000). In this set of experiments, the colorimetric reaction was carried out using SG blue (Sk-4700), by Vector Laboratories.

### Statistical analysis

The images of stained tissues were captured on a BX51 Olympus microscope using a 20x and 40x objectives with an Olympus DP70 digital camera controlled by analysis software (Soft Imaging System). Image acquisition was performed with the following fixed color settings: red = 0.36, green = 0.92, blue = 0.85, offset = 504, and exposure time of 6.8 milliseconds. At least 10 sections were analyzed from each mouse (3 HFD diabetic mice and 3 RD control mice). Counts were performed on a standardized area of 200 μm x 300 μm. The one-way analysis of variance (ANOVA) test was used to analyze difference between experimental groups. A probability level of .05 indicates significance.

### *In situ* hybridization

*In situ* hybridization for chemokine receptor CXCR4 mRNA was performed using digoxigenin labeled riboprobes. Wild-type 8 weeks-old male C57BL/6 (Jackson Laboratories) HFD induced diabetic and RD non diabetic DRGs were rapidly removed, embedded in OCT compound (Tissue Tek, Ted Pella, Inc., Redding, CA) and frozen. Sections were cut serially at 12 μm. The CXCR4 probe was generated as described previously [19,20]. Briefly, the rat CXCR4 cDNA was cloned as described [19,20] and a 870-bp *Sac*I/*Cla*I fragment from CXCR4/pcDNA3.1 construct was used as CXCR4 riboprobe, which can cross-react with mouse CXCR4. The template was linearized with XbaI to generate an antisense probe by using SP6 polymerase. The sense probe was linearized with HindIII by using T7 polymerase. *In situ* hybridization for CXCR4 mRNA was performed by using digoxigenin-labeled riboprobes (Roche Applied Science), as previously describe [19,20].

### Statistical analysis

The images of stained tissues were captured on a BX51 Olympus microscope using a 20x and 40x objectives with an Olympus DP70 digital camera controlled by analysis software (Soft Imaging System). Image acquisition was performed with the following fixed color settings: red = 0.36, green = 0.92, blue = 0.85, offset = 504, and exposure time of 6.8 milliseconds. At least 10 sections were analyzed from each mouse (3 HFD diabetic mice and 3 RD control mice). Counts were performed on a standardized area of 200 μm × 300 μm. Experiments were carried out in duplicates. The one-way analysis of variance (ANOVA) test was used to analyze difference between experimental groups. A probability level of .05 indicates significance.

## Discussion

CXCR4/SDF-1 signaling has been implicated in the pathogenesis of neuropathic pain in a subset of animal models as illustrated in previous reports from our own and other laboratories [19,20]. Importantly, CXCR4 expression is increased in a peripheral nerve microarray analysis from diabetic patients with progressive diabetic neuropathy [22], implicating CXCR4/SDF-1 signaling in the pathogenesis of diabetic neuropathy in humans. The present studies were designed to investigate a potential role for CXCR4/SDF-1 signaling in the pathogenesis of PDN. Indeed, we observed that the specific CXCR4 antagonist AMD3100 reversed PDN in two animal models of type II diabetes indicating that ongoing CXCR4/SDF-1 signaling is necessary for the manifestation of PDN. Furthermore, SDF-1 increased [Ca2+]i in DRG neurons from diabetic mice to a greater extent than in normal mice, suggesting increased CXCR4 receptor signaling in DRG neurons in the context of PDN. SDF-1 has also been shown to promote inflammatory cell infiltration into the DRG [10,30]. Indeed, we observed increased SDF-1 expression in DRG neurons and a massive influx of inflammatory cells into the DRG of diabetic animals that may have important implications for the generation of pain behaviors under these circumstances. Overall these results suggest that CXCR4/SDF-1 signaling plays a critical role in the pathogenesis of PDN by inducing excitatory effects on DRG neurons and recruiting inflammatory cells into diabetic DRG.

The present studies demonstrate for the first time that excitatory CXCR4/SDF-1 signaling in DRG neurons plays a critical role in the pathogenesis of PDN. Previous studies from our laboratory and others have demonstrated that SDF-1 and other chemokines can directly excite DRG neurons [20,25,29,35,36]. The pronociceptive effects of chemokines and their receptors include the induction of hyperexcitability of neurons through transactivation of transient receptor potential cation channel subfamily V member 1 (TRPV1) and other ion channels [37,38]. This process has been demonstrated on cultured and previously injured adult DRG sensory neurons [37,38]. We have now demonstrated that the specific CXCR4 antagonist AMD3100 reversed PDN in two animal models of type II diabetes indicating that ongoing CXCR4/SDF-1 signaling is necessary for the manifestation of PDN. As activation of CXCR4 chemokine receptors expressed by DRG neurons produces excitation [18], it is likely ongoing activation of CXCR4 receptors by SDF-1 released within the DRG contributes to the ectopic excitability of these neurons and produces AMD3100 reversible tactile hyperalgesia.

[Ca2+]i imaging experiments clearly demonstrated increased calcium mobilization in response to SDF-1 in cultured diabetic DRG neurons compared non diabetic control animals. Our laboratory and others have previously shown that activation of CXCR4 receptors by its ligand SDF-1 excites DRG neurons. Increased [Ca2+] i can be used as a readout of DRG neuron hyperexcitability in animal models of neuropathic pain [20,25,36]. Therefore, these data are consistent with the possibility that CXCR4/SDF-1 signaling plays a critical role in the pathogenesis of PDN by inducing excitatory effects directly on DRG neurons. Additionally, these data provide evidence of a considerable degree of CXCR4 receptor upregulation in acutely cultured diabetic DRG neurons. Interestingly, however, we actually observed a decline in CXCR4 gene transcriptional activity *in vivo* in diabetic CXCR4-eGFP mice. These results appear to contradict one another. However, an explanation was provided by further studies using *in situ* hybridization to detect CXCR4 mRNA expression in the DRG. We observed two types of CXCR4 expressing neurons in the DRG of normal mice. One population expressed very high levels of CXCR4 mRNA and the other group expressed lower levels. In diabetic mice we observed that the population of neurons that expressed very high levels of CXCR4 receptors was greatly reduced. In contrast the total number of neurons expressing CXCR4 receptors was increased. It is likely that we are only able to detect the population of neurons with high levels of CXCR4 expression using CXCR4-eGFP transgenic mice. This would explain why the number of CXCR4-eGFP cells is found to be reduced in diabetic CXCR4-eGFP transgenic mice, but we detect an increased number of cells expressing functional CXCR4 receptors using *in situ* hybridization experiments and [Ca2+]i imaging assays. The reason for this overall shift in the pattern of DRG neurons expressing CXCR4 receptors in diabetic mice is elusive at this point. However, we did note that DRG neurons from diabetic mice exhibited greatly increased levels of SDF-1.

We have demonstrated that SDF-1 can be released from DRG neurons [10,21]. Hence SDF-1 would be in a position to activate CXCR4 receptors expressed within the DRG of diabetic mice, which would be predicted to increase their excitability. Therefore it is possible that up-regulated expression of excitatory CXCR4 signaling within the DRG might be one factor driving increased excitability of these neurons in PDN.

Overall, these results are particularly interesting in light of recent publications using gene arrays to profile sural nerve samples from PDN patients, which reported that CXCR4 chemokine receptor expression was up-regulated in these samples [22]. Hence, the data from both mice and humans suggests the presence of up-regulated excitatory CXCR4 signaling by DRG neurons in association with PDN. These observations raise questions that now need further investigation, for example to determine which subtypes of sensory neurons are the major elements responsible for SDF-1/CXCR4 mediated neuropathic pain in diabetes.

During embryonic development DRG sensory neurons differentiate into three primary subtypes: nociceptors, mechanoreceptors and proprioceptors. In the DRG, these are characterized by the expression of the neurotrophin receptors TrkA, TrkB and TrkC, respectively [39]. During perinatal development nociceptors further differentiate into peptidergic (CGRP positive) and non-peptidergic (IB4 positive) [26]. We demonstrated that high levels of CXCR4 receptors, as determined using CXCR4-eGFP mice, were selectively expressed by IB4 positive nociceptive neurons. This observation is of particular interest in light of prior publications demonstrating that peripheral inflammation selectively increases TRPV1 function in IB4 positive sensory neurons in mouse models of neuropathic pain [40,41]. The enhanced responsiveness of IB4 binding neurons in the setting of inflammatory hyperalgesia could be mediated by CXCR4/SDF-1 signaling and be relevant in the pathogenesis of PDN. Our *in situ* hybridization studies also revealed the existence of a second population of DRG neurons, which express lower levels of CXCR4 receptors although we did not identify the properties of this neuronal subtype. Nevertheless, excitatory effects of CXCR4 activation in these neurons may also contribute to pain in diabetic mice.

In addition to their pronociceptive effects on DRG neurons chemokines and their receptors are important in the recruitment of leukocytes. The observed recruitment of numerous inflammatory cells into diabetic DRG suggests a critical role for inflammation in the pathogenesis of PDN. Immune cells can certainly secrete numerous algogenic factors, which may also have direct excitatory effects on DRG neurons or may sensitize DRG neurons to the excitatory effects of SDF-1 or other mediators [10,14]. SDF-1 has been shown to promote inflammatory cell infiltration into the DRG [10,30]. Hence SDF-1 released from diabetic DRG neurons may well influence the influx of inflammatory cells into DRG which may have important implications for the generation of pain behaviors under these circumstances.

The role of inflammation in the pathogenesis of PDN has been proposed elsewhere [42]. Specifically, inflammatory cytokines such as interleukins IL-6, IL-2, and tumor necrosis factor-α (TNF-α) are elevated in hyperglycemia, suggesting that a chronic low grade inflammatory state exists in patients with diabetes [43,44]. It has been observed that diabetic patients with higher plasma levels of TNF-α have a greater risk of developing PDN [43,45,46]. Moreover, compared with diabetic patients who have painless neuropathy those with PDN have been shown to have higher levels of C-reactive protein, considered to be a biomarker for any inflammatory process [47]. Recently, inflammatory mediators, including TNF-α, interleukin-1, 6, 13 and 17, chemokines such as MIP-1α, RANTES and fractalkine, have been found to be increased in the DRG of Zucker diabetic fatty (ZDF) rats, an established model for type II diabetes [48]. Additionally, molecules generated as the result of hyperglycemia including Advanced Glycolation End Products (AGE) and their receptor (RAGE) are considered important in the pathogenesis of PDN [49-52] and have been implicated in signaling pathways that induce transcription of proinflammatory genes [51]. In particular, chemokine expression is one important downstream target for both TNF-α and RAGE signaling. The present study provides additional support for a role of inflammation in the pathogenesis PDN providing a novel theoretical concept and also a new therapeutic target for the treatment of PDN.

In conclusion, these studies establish CXCR4/SDF-1 chemokine signaling as a new candidate responsible for the manifestation of PDN. The data suggest a mechanism as to how ongoing sensory neuron excitability could be increased in order to induce and maintain neuropathic pain in diabetes. This concept is that chemokine signaling has a fundamental role in in producing increased excitability of sensory neurons during the transition from physiological to neuropathic pain in diabetes, as well as its subsequent maintenance. This is something that has not been previously appreciated with respect to PDN. Furthermore, SDF-1 could potentially play a crucial role in both recruiting inflammatory cells and producing direct excitatory effects on DRG neurons thereby coordinating the interaction between peripheral neurons and immune cells. Hence, targeting CXCR4/SDF-1 signaling may lead to advanced therapeutic approaches for the treatment of PDN. This novel and more targeted therapeutic intervention has advantages over existing and currently available therapeutic approaches which aim only to reduce symptoms generally by suppressing neuronal activity and are not particularly effective. In contrast, modulation of proalgesic chemokine signaling provides an opportunity for disease modification by inhibiting inflammatory process and neurobiological alterations that support the development of persistent pain.

## Competing interests

The authors declare that they have no competing interests.

## Authors’ contributions

DMM prepared the manuscript, performed Von Frey behavioral studies, calcium imaging studies, immunohistochemical labeling, confocal analysis and statistical analysis, AB performed calcium imaging studies, AS performed immunohistochemical labeling, CF helped with Von Frey behavioral studies, DR cultured DRG neurons for calcium imaging studies and in situ hybridization, RJM supervised the project and help writing the manuscript. All authors read and approved the manuscript.
